# Implementation of Global Action Plan On the Public Health Response to Dementia (GAPD) in Sub-Saharan Africa: Comprehensive Reviews

**DOI:** 10.1192/j.eurpsy.2023.877

**Published:** 2023-07-19

**Authors:** L. Ssenyonjo, I. Ddumba

**Affiliations:** 1 Research & Innoviation; 2 Nursing, Victoria University, Kampala, Uganda

## Abstract

**Introduction:**

Despite the fact that, age is a strongest know risk factor for onset of dementia, and developing countries are projected to have highest number of ageing population, few national dementia strategies have been put in place to address this impending scourge. In 2017, World Health Organization(WHO) released and called for countries to adapt and contextualize the Global Action Plan on the Public health response to dementia, few Sub-Saharan countries have slowly adopted plan. The outcome of the unprecedented increase populations with dementia will be immense. The substantial increase in morbidity and mortality pose a threat to the over stretched health care system and undermine the potential to achieve sustainable development goal (SDGs).

**Objectives:**

Understading the implementation of Global Action Plan on the Public health response to dementia in the developing countries

**Methods:**

This paper is a view of published and grey literature relevant to Global Action Plan On the Public Health Response to Dementia (GAPD) in sub-Saharan Africa. The overall approach to the review had an exploratory and inductive focus. Articles were categorized around a guiding conceptual framework. Like; A description of structural arrangements and content of national dementia strategy development and normative underpinnings within policy frameworks

**Results:**

Nearly all countries within the SSA hadn’t developed the national dementia strategy plans. Countries like South African, Ghana, Kenya and Ethiopia had drafts of national dementia strategy, though not yet operationalized. Few countries highlighted some of the parallel targets of GAPD within their national mental health policy and strategy, but it was not comprehensive. Countries where Civil societies that advocate/champion dementia activities were strongly presently were more likely to possess a draft of GAPD.

**Conclusions:**

Although there some initiatives for different countries to develop national strategy for dementia plans, there are gaps in the extent of engagement of different stakeholders and how these strategies will be operationalized may limit the impact on addressing the escalating burden of dementia in Sub-Saharan Africa.

**Disclosure of Interest:**

None Declared

